# Synthesis of Ag-Doped Tetrahedral Amorphous Carbon Coatings and Their Antibiofilm Efficacy for Medical Implant Application

**DOI:** 10.3390/nano14121017

**Published:** 2024-06-12

**Authors:** Davoodbasha MubarakAli, Sung-Min Kim, Yu-Been Ko, Jung-Wan Kim, Young-Jun Jang, Sang-Yul Lee

**Affiliations:** 1 Center for Surface Technology and Applications, Korea Aerospace University, Goyang 10540, Republic of Korea; mubinano@gmail.com; 2 School of Life Sciences, B.S. Abdur Rahman Crescent Institute of Science and Technology, Chennai 600048, India; 3 Crescent Global Outreach Mission (CGOM), B.S. Abdur Rahman Crescent Institute of Science and Technology, Chennai 600048, India; 4 Heat and Surface Technology R&D Department, Korea Institute of Industrial Technology (KITECH), Incheon 21999, Republic of Korea; sungminkim@kitech.re.kr; 5 Division of Bioengineering, Incheon National University, Songdo, Incheon 22012, Republic of Korea; 202121115@inu.ac.kr (Y.-B.K.); kjw5864@inu.ac.kr (J.-W.K.); 6 Surface Technology Division, Korea Institute of Material Sciences, Changwon 51508, Republic of Korea; yjjang@kims.re.kr

**Keywords:** tetrahedral amorphous carbon, Ag-taC, coating, anti-biofilm, *Staphylococcus aureus*

## Abstract

Tetrahedral amorphous carbon (taC) is a hydrogen-free carbon with extensive properties such as hardness, optical transparency, and chemical inertness. taC coatings have attracted much attention in recent times, as have coatings doped with a noble metal. A known antimicrobial metal agent, silver (Ag), has been used as a dopant in taC, with different Ag concentrations on the Ti64 coupons using a hybrid filtered cathodic vacuum arc (FCVA) and magnetron sputtering system. The physiochemical properties of the coated surface were investigated using spectroscopic and electron microscopy techniques. A doping effect of Ag-taC on biofilm formation was investigated and found to have a significant effect on the bacterial-biofilm-forming bacteria *Staphylococcus aureus* and *Pseudomonas aeruginosa* depending on the concentration of Ag. Further, the effect of coated and uncoated Ag-taC films on a pathogenic bacterium was examined using SEM. The result revealed that the Ag-taC coatings inhibited the biofilm formation of *S. aureus*. Therefore, this study demonstrated the possible use of Ag-taC coatings against biofilm-related complications on medical devices and infections from pathogenic bacteria.

## 1. Introduction

Generally, surfaces coated with appropriate materials for medical implants are enormously susceptible to prosthetic failure, viz., erosion, hardness, and chemically reactive wettability, eventually contributing to biofilm formation. Biofilm formation on medical devices has a significant impact on health and post-surgical complications. There are plenty of materials that have been coated on the surfaces of metals and metal alloys to make them biocompatible, for instance the coating of hydroxyapatite along with silver (Ag) and copper (Cu) [1,2]. It has been found that the physiochemical properties of coatings at the interface robustly hinder biological features. Formulating the agent to be incorporated on the surface is essential to facilitate a specific surface with a biological reaction by tuning the surface chemistry and studying factors which have an influence on it [3]. Coating materials exhibiting a hydrophobic surface are more significant than hydrophilic surfaces on general medical devices and implants [4]. 

In recent times, tetrahedral amorphous carbon (taC), a type of carbon material that is interconnected with sp^3^ sites, has attracted much attention for coatings due to its high fraction of about more than 60% sp^3^ carbon [5], its hardness [6], chemical inertness [7], electrochemical activity [8], wear resistance [9], thermal stability [10], and biocompatibility [11]. As per the ISO standard 20523:2017, a hardness of >40 GPa is needed for carbon t be considered taC [12]. Doped DLC films can change the hardness and surface free energy [13]. However, hardness is not just considered for improving tribological behaviors [14]. The enhanced physiochemical properties of taC can be attained by doping it with metal and adjusting the sp^3^/sp^2^ ratio [15,16]. In addition, superhydrophobicity, super hydrophilicity, and superoleophilicity for oil–water separation can also be tuned [17]. Notably, taC has been used in a broad range of analyte detection applications, including H_2_O_2_ sensing [11], the detection of pharmaceutical molecules [15], and involvement in neural signal and gene expression [18,19]. 

Although taC has exhibited a broad range of applications autonomously, its properties are superior when doped with metals or gases as dopants. Silicon and Molybdenum are the ideal dopants for tribological applications among the different metal dopants studied [20]. Minimizing the interactions between Copper and Carbon during coatings can retain the carbon film’s transparency rate and eventually increase the surface properties [21]. Specifically, Ag metal created a significant impact among others as standalone, composite, or doped materials, and increased the surface hydrophobic properties [22], electron field emission properties [23], and biocompatibility [24]. Furthermore, Ag nanoparticles are used to dope on the surface of graphene oxide for enhanced catalytic properties [25].

Sputtering, pulsed laser deposition, electron cyclotron wave resonance, and physical vapor deposition can all be used to make taC films [26,27,28] since these methods have certain limitations in routine protocols due to expensive coating amenities and a relatively lower sp^3^ bonding content [29]. Very recently, the FCVA method has been adopted to obtain a taC coating that is highly efficient in film formation in terms of size and uniform distribution, and its mechanical and chemical properties are also significant. The FCVA uses the solid carbon target up to the high carbon ion energy of 10 to 30 eV. The FCVA method involves the arc evaporation of carbon and the formation of more than 70% charged carbon ions with an energy of >25 eV [30]. Similarly, magnetron sputter sources are a reliable and cost-effective method. The combination of a magnetron sputter source with plasma source ion implantation is called magnetron plasma source ion implantation. In this study, Ag was doped on a taC coating, and the tribological characteristics of the resulting Ag-taC were investigated for their antibiofilm efficacy on the implant material against pathogenic biofilm-forming bacteria. 

## 2. Materials and Methods 

### 2.1. taC Coating Preparation

**Figure 1:**
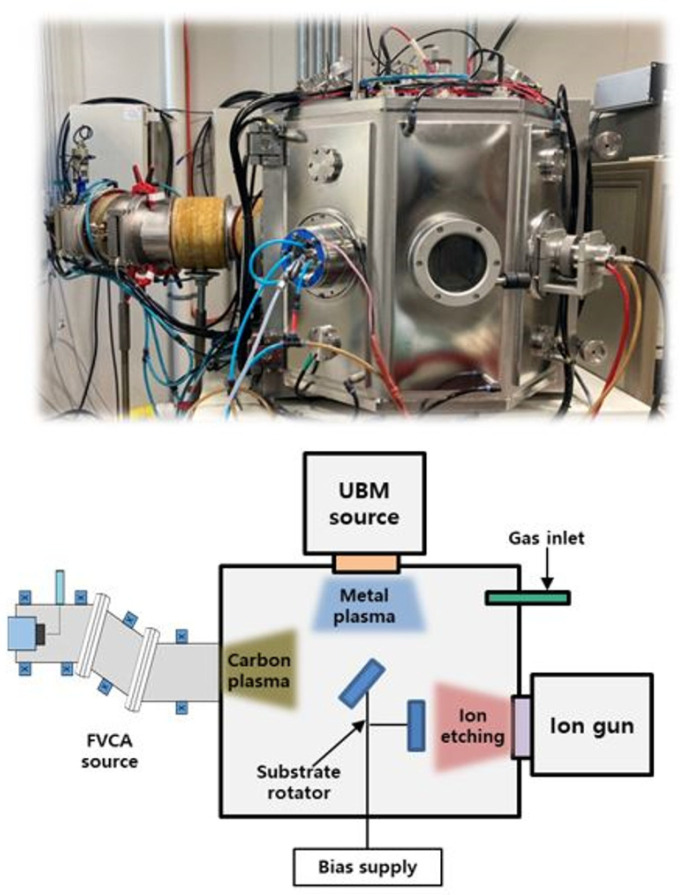
Schematic illustration of the hybrid coating system: FCVA and magnetron sputtering coating system for taC and Ag-taC preparation.

The taC coating was synthesized using a 45° bent-type filter in an FCVA system, which was composed of a linear ion source, an unbalanced magnetron sputter (UBM), and a carbon arc. Figure 1 shows a schematic diagram of the hybrid coating system used in this work. A detailed description of the equipment and synthesis methods has been reported previously [31]. Briefly, single crystalline silicon (1 0 0) wafers of 20 mm × 20 mm × 0.5 mm and Ti64 of 20 mm × 20 mm × 3 mm were used as substrates. Prior to deposition, Ar plasma at 2.0 × 10^−1^ Pa was used to etch the substrate surface for 20 min using the linear ion source. A carbon target 50 mm in diameter and of 99.99% purity was used as a carbon source. The deposition conditions for FVAC were duct bias, 10 V; arc current, 60 A; substrate bias, −30 V; and working pressure, 3.0 × 10^−2^ Pa. UBM sputtering (Ar gas flow: 15 sccm) was simultaneously discharged during ta-C coating for Ag doping and sputtering conditions with a pressure of 3 × 10^−3^ Pa, Ar gas flow rate of 25 sccm, and a direct current (DC) sputtering power of 30, 50, and 70 W for controlling the Ag content in the taC coatings. The thickness of all films was about 200 nm.

### 2.2. Surface Characterizations

X-ray photoelectron spectroscopy (XPS, K-alpha, Thermo Fisher Scientific Inc., London, UK) with Al Kα radiation aided the chemical bonding state analysis and sp^2^/sp^3^ characterization of the taC and Ag-taC coating films. The conditions for the XPS analysis are an X-ray source with variable spot size (30–400 µm in 5 µm steps) and energy resolution about FWHM ≤ 0.5 eV for the Ag 3d_5/2_ and angle-resolved at 0–70° and base pressure of 4.8 × 10^−9^ bar. Raman spectroscopy (Horiba Jobin Yvon, Kyoto, Japan; λ = 514 nm) aided in the structural analysis of the taC coating film. The following conditions were used: laser power of <1 mW; spot size of 1 µm; and grating of 600 gr.mm^−2^. Using the Gaussian fitting function, the measured Raman single spectrum was fitted to the centers of 1350 cm^−1^ and 1580 cm^−1^ wavelengths for the D peak and G peak, respectively. The coating morphology and structural evolution concerning Ag content were investigated by field-emission scanning electron microscopy (FE-SEM, JSM-7800F, JEOL Ltd., Tokyo, Japan, operated at 5 keV). Atomic force microscopy (AFM, Park NX10, Park Systems, Suwon, Republic of Korea) measured the average roughness by non-contact measuring 10 points with a scan area of 128 µm^2^ using the specific cantilever (XE-100, Park System, Suwon, Republic of Korea). A cross-section image was also analyzed to understand the taC thickness using SEM. Energy dispersive spectroscopy (EDS) mapping was also performed to determine the Ag distribution on the taC. The surface roughness of the coated substrates was evaluated using a contact angle (CA) measurement by the Sessile-drop method using deionized water with a 5 μL volume (SJ-410, Mitutoyo, Sakado, Japan). 

### 2.3. Biofilm Formation on taC and Ag-taC

The taC and Ag-taC coated on the Ti64 were sterilized by washing with 70% ethanol for 20 min, followed by UV irradiation on both sides of the samples for 16 h. Biofilm-forming Gram-negative and Gram-positive bacteria, *Pseudomonas aeruginosa* and *Staphylococcus aureus,* respectively, were precultured in Tryptic Soy Broth (TSB) containing 1.7% pancreatic digest of casein, 0.3% papain digest of soybean, 0.25% dextrose, 0.5% sodium chloride, and 0.25% dipotassium phosphate (Difco, Atlanta, GA, USA) incubated at 37 °C for 16 h with shaking at 200 rpm (SW-905G, Sangwoo Tech, Seoul, Republic of Korea). The preculture was diluted to 10^7^ CFU.mL^−1^ concentration using TSB for *P. aeruginosa* and TSB with 1% glucose for *S. aureus*. The coating of Ti64 coupons with taC and Ag-doped taC at various doping percentages was carried out in Petri-dishes (10035, SPL, Pocheon, Korea), and 4 mL of the bacterial suspension (10^7^ CFU.mL^−1^) was added to the dishes. The samples were incubated for 24 h at 37 °C with no shaking. Afterward, they were carefully rinsed twice in 0.85% NaCl with gentle shaking (80 rpm) for 5 min. One percent of crystal violet solution dissolved in 20% ethanol was added to the samples until they were submerged, and they were then incubated for 10 min. After that, they were washed three times with deionized water and dried. Four milliliters of dimethyl sulfoxide (DMSO) were added to the samples and they were incubated for 10 min for de-staining and absorption at 570 nm using a UV/Vis spectrophotometer (UV-1280, Shimadzu, Kyoto, Japan). Further, bacterial biofilm formation on the coated and uncoated films was investigated using SEM (JSM-7800F, JEOL Ltd., Tokyo, Japan) analysis. 

## 3. Results and Discussion

**Figure 2:**
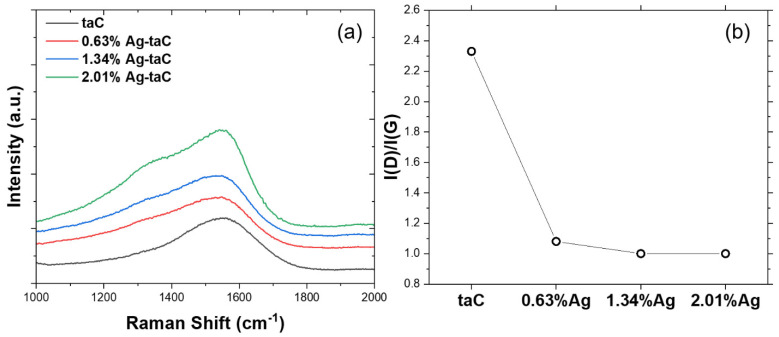
Raman spectra of Ag-taC coatings (**a**); and I(D)/I(G) ratio (**b**).

In general, Raman spectroscopy is an effective nondestructive tool, and the data are greatly relevant to carbon-material-related bonds such as sp^2^ and sp^3^ and are used to characterize the structure of DLC [32]. It was used to investigate the quality of fabricated taC films. Figure 2a shows the Raman spectra of the taC, and various concentrations of Ag-taC coating materials were deposited on the Ti64 alloy. A broad peak at 1555 cm^−1^ indicates the signature peak for amorphous carbon. It is indicated that doping the Ag dopant in the film leads to a widening of the peak at different degrees.

In an earlier study, the roughness of the Ni-Cu alloy substrate was 272 nm at the accelerator voltage, and the deposition time was 2200 V and 30 min, respectively, which generated a DLC thin film with a thickness of 121 nm [33]. The deposition time of the present study was increased three-fold to attain a uniform thickness of 200 nm using FVCA. The characteristic peaks in the 1300–1400 cm^−1^ range are Gaussian D peaks, and those in the 1500–1600 cm^−1^ range are Gaussian G peaks. As a matter of fact, the D peak is highly relevant to a disordered structure, whereas the G peak represents the interconnected hexagonal carbon rings. In addition, the D and G peaks are assigned to a 3D atomic organization composed of strongly interlinked atoms, and 2D atomic orientation, respectively [34,35]. 

Interestingly, a Gaussian D peak was formed at 1350 cm^−1^ in the Ag-taC at a concentration of 2.01 at. %. This could be due to the doping of Ag at the highest concentration causing the extra peak along with the typical taC peak at 1555 cm^−1^. The breathing modes of sp^2^ carbon atoms in the ring exhibited in the D peak at 1360 cm^−1^ were reported elsewhere [36]. Based on the asymmetric Raman band, two peaks correspond to the G peak were observed at 1570 cm^−1^ and the D peak at 1360 cm^−1^. These peaks were formed due to the vibrations and breathing modes of sp^2^ carbon in the aromatic rings and chains [37]. Based on Figure 2b, the ID/IG ratio was compared with taC and Ag-doped taC, showing that taC had the highest position at 2.3, and Ag-taC was shown at the lowest, while the other variants had rather similar positions, such as 1.1. Notably, the addition of Ag dopant at the highest concentration (Ag-taC, 2.01 at. %) showed an increased G position compared to that of Ag-taC (0.63 at. %) and Ag-taC (1.34 at. %), which showed 1547 cm^−1^ and 1545 cm^−1^, respectively. The deconvolution of the Raman spectra showed the ID/IG ratio’s integrated intensity, which is considered an ideal parameter to confirm the carbon’s thin film property [38].

**Figure 3:**
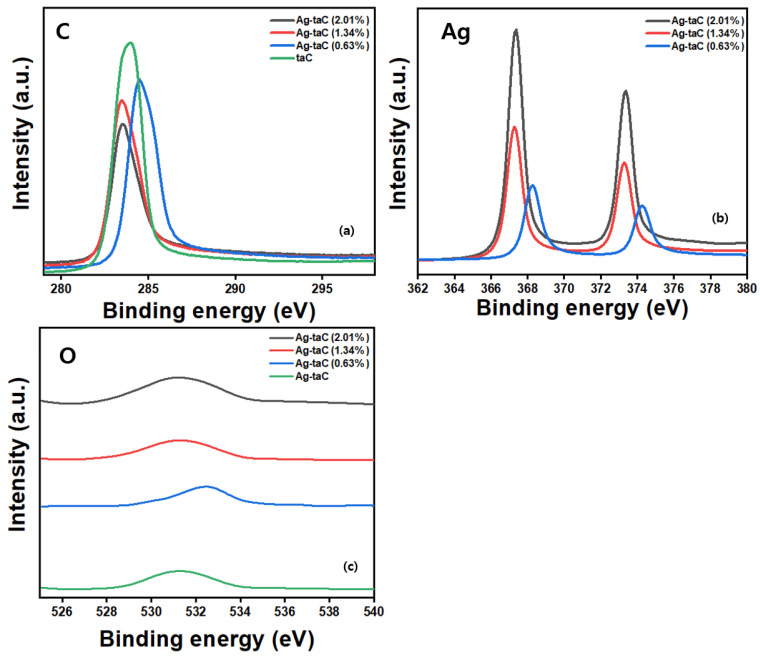
XPS spectra of the taC and Ag-taC coatings demonstrating the typical binding energy for C (**a**), Ag (**b**), and O (**c**).

The XPS technique was used to assess the chemical bonds of the taC film fabricated at various sputter powers (30 W, 50 W, and 70 W). After the coating was etched with Ar gas, high-resolution spectra of C1s, Ag3d, and O1s were taken from pure taC and Ag-taC (Figure 3). Figure 3a is mostly composed of sp^3^, which indicates the tetrahedral amorphous carbon in the film. It shows the highest intensity, indicating pure taC on the film. Notably, when a low concentration of Ag doping was used (0.63 at. %), the spectra revealed a stretched region near 283 eV, indicating the emergence of Ag–C and/or Ag–C–H bonds. This finding was likely attributed to the bonding between carbon atoms and the deposited Ag. The aerial fraction of Ag–C or Ag–C–H bonds increased when the Ag composition in the film increased. In addition, the fraction of sp^3^ decreased sustainably with an Ag dopant increase. 

The XPS spectra of O1s also indicated a peak shift observed after a decrease in the C–O–C bonds. Nevertheless, the most critical observation in O1s spectra was the decrease in intensity with an increase in Ag fraction (Figure 3b). The typical C1s peak deposited at a 30 W was deconvoluted into four sub-peaks at 283.86 eV, 284.58 eV, 283.55 eV, and 283.57 eV, which corresponded to sp^2^–C, sp^3^–C, C–O, and C–Ag bonds, respectively, as shown in Figure 3a. This result corroborates previous reports stated and reported elsewhere [36,39,40]. In Figure 3c, the typical O1s spectra can be superposed by peaks at 531.38 eV, 532.43 eV, 531.45 eV, and 531.55 eV, belonging to C–O, O–C=O, C–O, and O–Ag bonds. The corresponding binding energy values are presented in Table 1. Ding et al. stated that oxygen pollution is due to the presence of the oxide niobium in film [36]. They reduced the oxygen impurity by altering the bias voltage conditions. A peak shift after a decrease in the C–O–C bonds was found where the intensity of the O1s spectra decreased with increasing Ag fraction.

**Figure 4:**
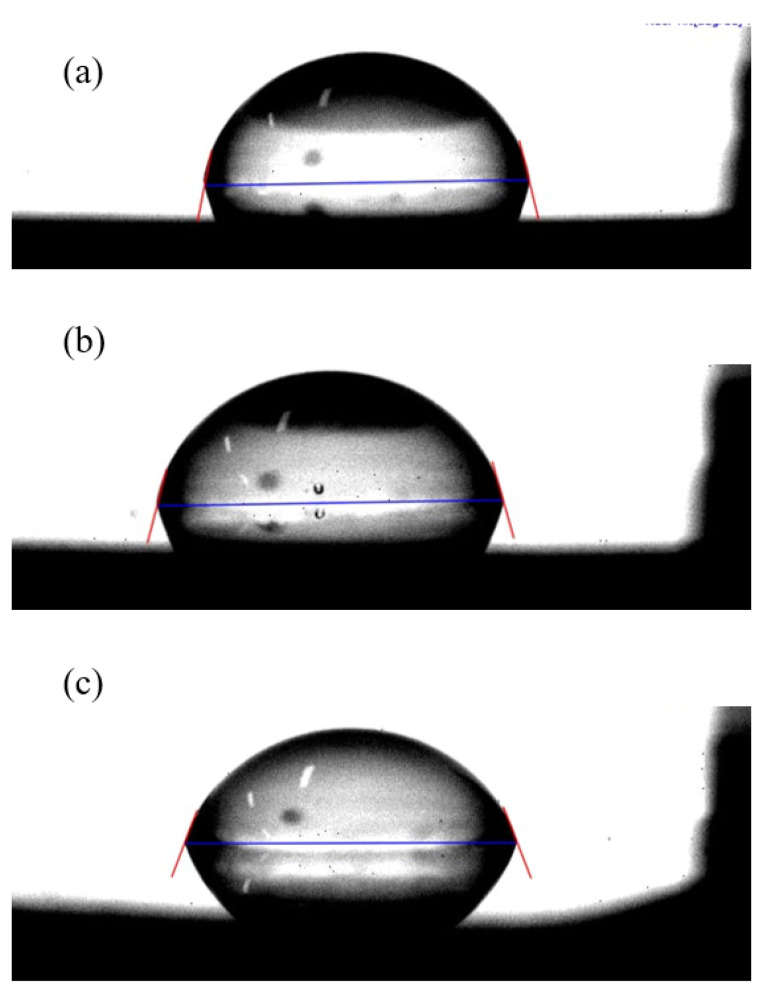
Represents the optical micrographs of droplets on taC and Ag-taC films deposited by different Ag-doping conditions: (**a**) 0.63 at. %; (**b**) 1.64 at. %, and (**c**) 2.01 at.%.

Figure 4 shows the profile of the hydrophobicity of a water drop and its contact angle on the taC and Ag-taC film surfaces. Generally, less than 30° indicates a low contact angle with high surface energy, whereas >75° indicates a high contact angle with low surface energy [41]. The CA measurement implies that the hydrophobicity of the Ag-doped taC films increased with Ag doping on their surfaces. The CA of taC was 64.2°; it had good wetting properties. Ag-taC exhibited 70.80°, 76.48°, and 79.43° based on Ag doping: Ag-taC (0.63 at. %), Ag-taC (1.64 at. %), and Ag-taC (2.01 at. %), respectively. Ag doping in taC film coatings induced hydrophobicity on the surface. Similarly, increased hydrophobicity was observed in DLC coatings on the substrate due to amorphous carbon thin films [42]. It is also demonstrated that Ag deposition on the surface of DLC films varies between 79 and 95° [33]. Comparatively, the hydrophobic surfaces were found to be more crucial than the hydrophilic ones for the medical implants. In an earlier report, the accumulation of Ag on the surface improved the hydrophobicity of the DLC films [33]. The variations in static CA on the coated surfaces were observed in relation to Ag doping. The data verified a decrease in surface energy, increasing sputtering power. 

**Figure 5:**
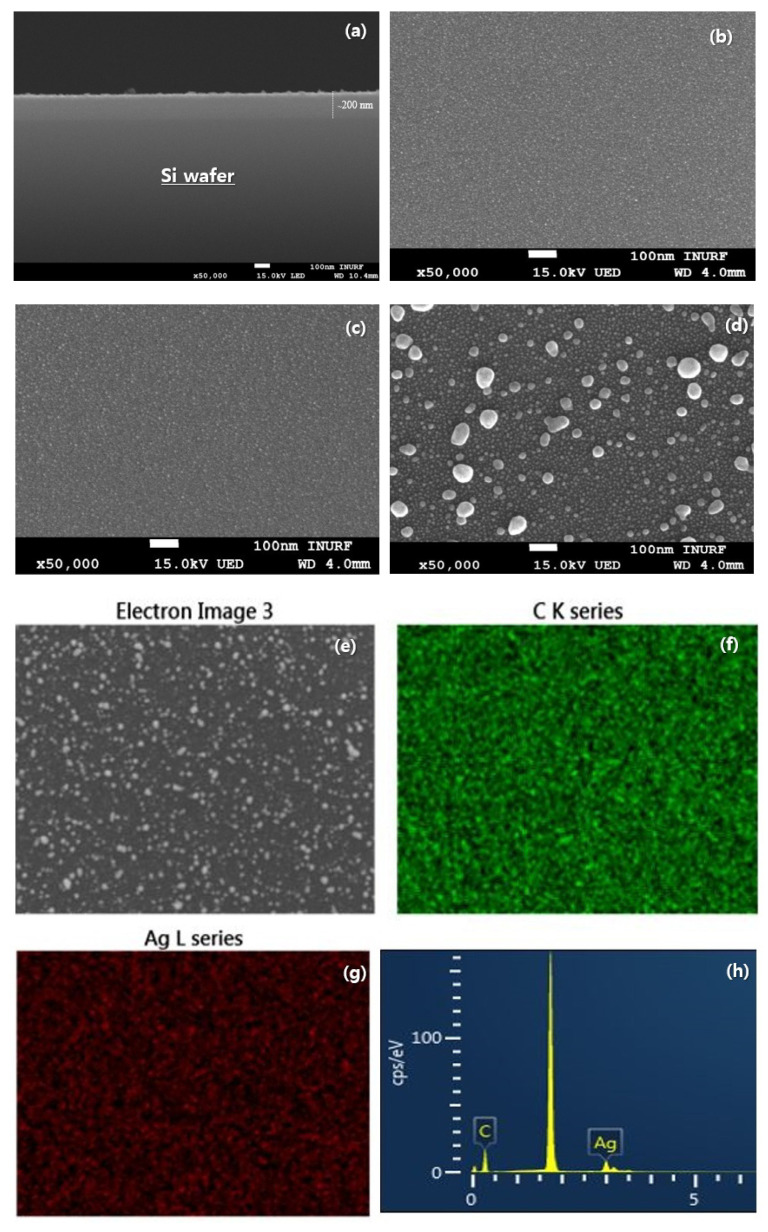
SEM data of Ag-taC coatings with representative sputtering power of 30 W, 50 W, and 70 W with a coating thickness 200 nm on Si wafer (**a**) 0.63 at. %, (**b**) 1.34 at. %, (**c**) 2.01 at. %, (**d**) Ag-taC were deposited on Si-wafer and prepared by cross-sectional cutting. (**e**) SEM with EDS data of Ag-taC (2.01 at. %) film showed the distribution of Ag-taC, predominantly. (**f**) EDS mapping showed carbon, (**g**) Ag, and (**h**) level of element percentage.

The surface morphology of the synthesized Ag-doped taC samples was characterized using FE-SEM, as shown in Figure 5. The images revealed distinct variations in the surface morphology as the Ag-doping concentration increased, achieved by varying the sputter power. For the Ag-taC (0.63 at. %) sample prepared with a sputtering power of 30 W, the SEM image displayed a slight increase in the size of the taC grains compared to the pure taC surface. The thickness of Ag-doped taC can be observed in Figure 5a. However, the overall surface remained relatively smooth and dense. Moving on to the 1.34% Ag-taC sample prepared with a sputtering power of 50 W, a more noticeable change in surface morphology was evident, as shown in Figure 5c. The taC results appeared more significant than those of both the pure taC and the 0.63% Ag-taC. For the 2.01% Ag-taC sample prepared with a sputtering power of 70 W, the SEM image revealed the most significant alteration in the surface morphology (Figure 5d). 

The observed variations in surface morphology with increasing Ag-doping concentration in the synthesized Ag-doped taC samples can be attributed to the combined effects of Ag composition and sputter power. As the Ag-doping concentration increases, the presence of Ag atoms within the taC matrix becomes more pronounced. It is presumed that the large, island-like grain observed predominantly consists of Ag. Thus, incorporating Ag atoms into the taC matrix can significantly influence the growth behavior of the grains and contribute to grain coarsening. The higher the sputter power, the higher the kinetic energy of the sputtered atoms, which enhances the mobility of the adatoms on the substrate surface during the deposition [43]. As a result, the adatoms have a greater tendency to diffuse and rearrange, increasing grain size in the deposited film. The EDS of Ag-doped taC (2.01 at. %) with a sputtering power of 70 W film showed the distribution of Ag in taC, predominantly (Figure 5e). EDS mapping showed C, Ag, and elemental percentages (Figure 5f–h), respectively. 

**Figure 6:**
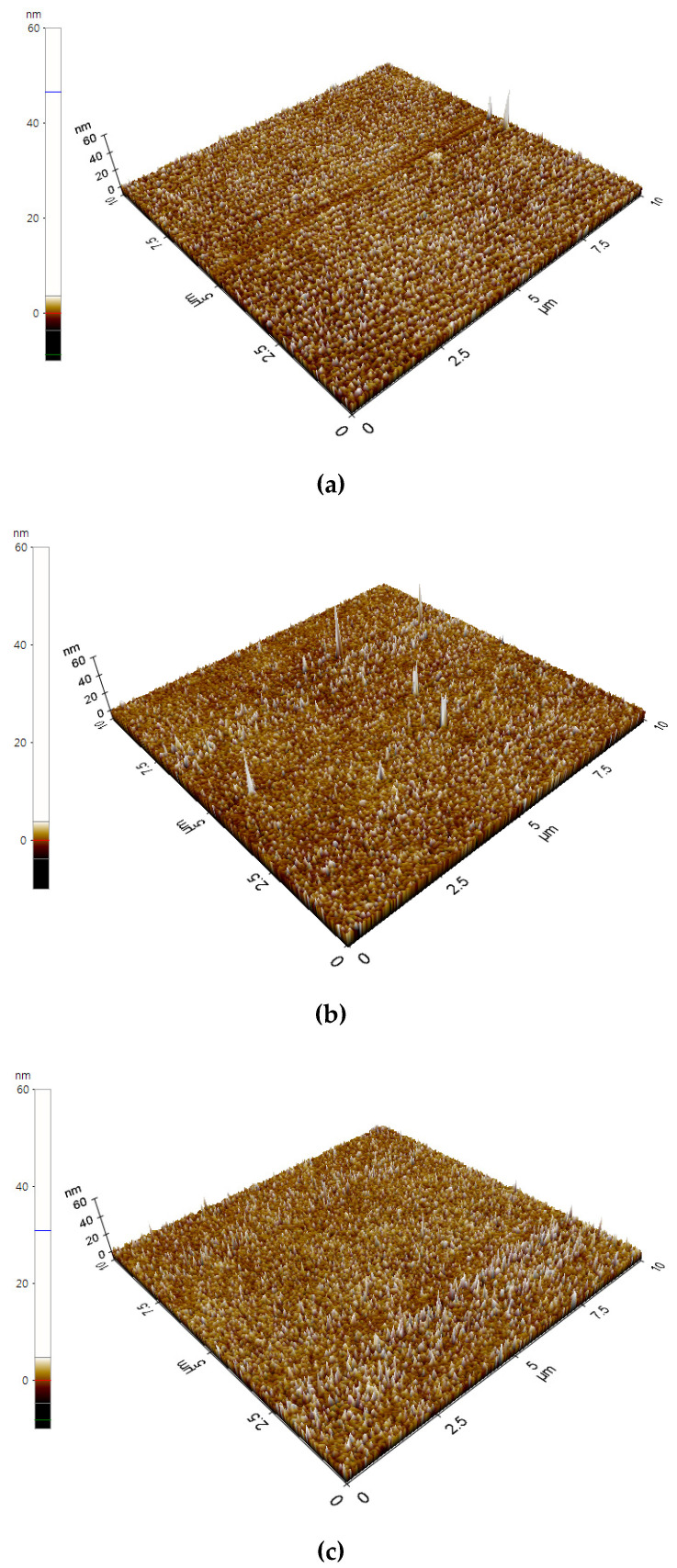
AFM showed surface roughness images of Ag-taC coatings 0.63 (at. %) (**a**), 1.34 (at. %) (**b**), and 2.01 (at. %) (**c**) with their average roughness (Ra) and root mean square (RMS) calculated as 1.4 nm and 1.89, 1.3 nm and 2.0, and 1.7 nm, and 2.4, respectively.

Figure 6a–c show the three-dimensional AFM surface topography of various Ag-doped taC as Ag-taC (0.63 at. %), Ag-taC (1.34 at. %), and Ag-taC (2.01 at. %) deposited on the Ti64 alloy at a bias voltage of −200 V. The surface roughness increased as the concentration of Ag increased in the film. The surface roughness of Ag-taC was found to be 1.4 nm, 1.3 nm, and 1.6 nm for Ag-taC (0.63 at. %), Ag-taC (1.34 at. %), and Ag-taC (2.01 at. %), respectively. The smoothing process is probably due to the ion bombardment effect. After doping with Ag, the RMS values of Ag-taC film increased by 1.8, 2.0, and 2.4. Ag ion doping causes an increase in surface roughness. Similarly, Ni ion doping increases the surface roughness based on the surface swelling phenomena that have been associated with the varied density and modified layer thickness discussed elsewhere [44]. In contrast, Ding et al. found that surface roughness declined with an increase in bias voltage from −100 V to −300 V [36]. They also stated that the impinging ions at high energy lead to an increase in atom mobility. Hence, they transform the sites from hillocks to dense structures and eventually form a smooth structure. Shen et al. found that the difference in deposition techniques might cause taC films to have a relatively rougher surface than amorphous carbon [45].

**Figure 7:**
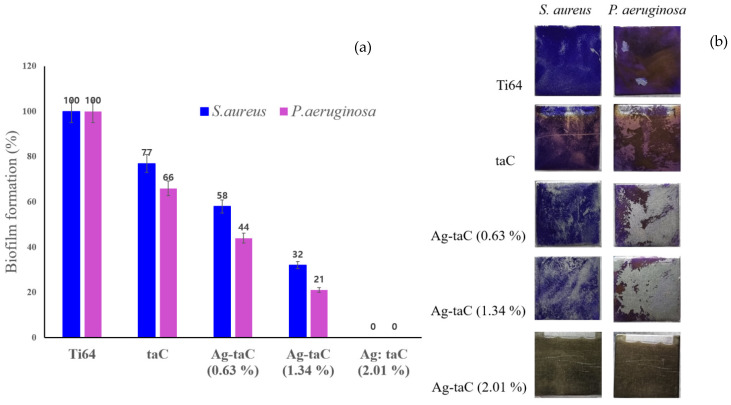
Biofilm formation on the taC and Ag-taC: the biofilm formation decreased sustainably as the con-centration of Ag increased on both *P. aeruginosa* and *S. aureus* (**a**); light microscope analysis on the surface of the coated coupons (**b**).

In the biofilm biomass quantification assay, Ag-taC showed good biofilm inhibitory activity against the clinically relevant pathogens *P. aeruginosa* and *S. aureus*. Figure 7a shows that the Ti64 has no biofilm inhibitory potential against the tested pathogens, *P. aeruginosa* and *S. aureus*. With the combination of taC, the inhibitory potential of Ti64 was increased up to 30 to 35%. Adding Ag to the composites further increases the potential of the nanocomposites against the biofilm formation of the test pathogens. From the results, it is clear that the Ag concentration is directly proportional to the biofilm inhibitory potential of the nanocomposites. At the maximum, Ag-taC with (2.01 at. %) Ag completely inhibited the biofilm formation of the test pathogens. The obtained quantitative results were further validated quantitatively with light microscope analysis on the surface of the coated coupons (Figure 7b).

*Staphylococcus aureus* and *Pseudomonas aeruginosa* are known to cause a range of infections in humans. They are considered clinically important pathogens because of their ability to cause severe diseases, their high prevalence in healthcare settings, and their ability to develop antibiotic resistance. Most importantly, both bacterial pathogens can form robust biofilms in healthcare settings, further complicating treatment and contributing to their pathogenicity [46,47]. Nanoparticles are already known to target bacterial growth through a different mechanism of action, and studies on nanoparticles against the biofilm formation of bacterial pathogens are on the rise [48]. Based on the results, adding Ag to taC is a possible reason for the enhanced biofilm inhibition in the test pathogens. In accordance with the findings, the Ag, Cu, and MgO nanoparticles synthesized have a similar effect on the biofilm formation of other pathogens, such as *E. coli, S. aureus,* and *P. aeruginosa* [2,49,50,51].

**Figure 8:**
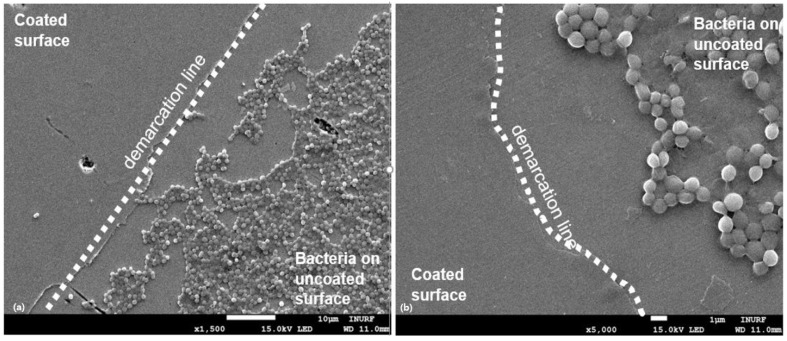
SEM image of Ag-taC on Ti64 showing the biofilm formation on the coated and uncoated sites, indicating that the coated site is free from the bacterial biofilm (**a**) and closure view of de-marcation line between coated and uncoated sites (**b**).

To support the evidence of biofilm inhibition on the surface of Ag-taC coatings, it was investigated using SEM studies (Figure 8). The demarcation line was clearly visible at the lowest magnification between the coated and uncoated surfaces. On the uncoated surface, 97% of the area was covered with biofilm caused by *S. aureus* (Figure 8a). Although the biofilm inhibition study was initiated with *S. aureus* and *P. aeruginosa*, the SEM study was focused only on *S. aureus* since the organism is considered a potential biofilm-forming bacterial pathogen and causes severity in hospital-related diseases such as nosocomial infection. It also develops multi-drug resistance [47]. At the highest magnification, *S. aureus* established an apparent growth on the uncoated surface by developing extracellular polymeric substances (EPS). The *S. aureus* mostly appears to be spherical, with a clump of cells. It is significant evidence that the cells are not only growing on the coated surfaces but also near the demarcation line on the uncoated surface, indicating that the effect of Ag-taC did not allow any cells to adhere near the coated surface (Figure 8b). This result corroborates with Streletskiy et al., who stated that bacterial adhesion differs with taC film coatings doped with Ag and N_2_ [52]. They found that bacterial proliferation was observed in taC: N coatings and found a gradual decrement in the bacterial population. It is stated that bacterial biofilm develops resistance to prosthetics and medical treatment and gradually decreases immunity [53].

**Figure 9:**
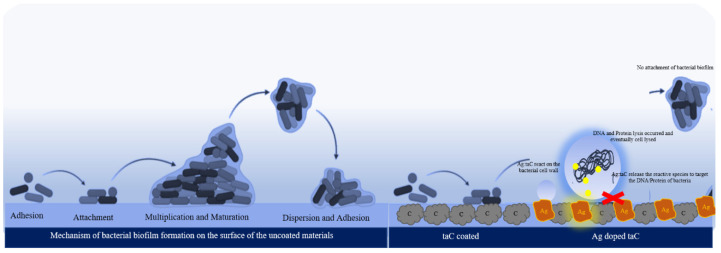
Mechanism of biofilm formation and biofilm inhibition by the Ag-doped taC.

The enhanced activity of the Ag-taC observed in the present work is assumed to be the synergistic effect of Ag with the taC structures, which will also be expected to further enhance the properties of the coatings, like wearability, long-term durability, and other properties related to the components of the resulting composite materials [54]. The findings of the present work are substantiated by the recent work of Streletskiy et al., wherein taC decorated with silver showed better antibacterial and antibiofilm properties against *S. aureus* [52]. In detail, the samples coated with Ti alone failed to inhibit the adherence of *S. aureus*, whereas the addition of Ag to Ti enhanced the antibacterial potential and reduced the adherence of bacterial cells to the coated surface. Moreover, the tested concentration of taC-Ag had no cytotoxicity toward fibroblast cells. The Ag-doped taC coatings directly impact the bacteria to overcome the biofilm-related implant failure. Furthermore, the mechanism of action in the coated film is illustrated by elaborating on the biofilm formation and inhibition by the coated surfaces (Figure 9). 

Generally, biofilm formation occurs initially by cell adhesion on the surface where the surface is wettable. The materials’ hydrophilic nature facilitates the cell’s migration and binding to the surface. Cells attach to the surface with a quantum of cells after cell adhesion. Due to the cell–cell communication process, the so-called quorum sensing mechanism, nearby cells are bound with attached biofilm. This strong attachment is facilitated by extracellular polymeric substances and trace proteins [55]. In addition, the attached biofilm multiplies and matures enough to adhere to the other sites to establish the biofilm, and eventually biocorrosion occurs and leads to graft rejection and secondary bacterial infections [2]. Many studies have been reported on the antibacterial effect of Ag nanoparticles on different types of bacteria that cause various illnesses [56]. Biofilm formation on medical devices and implants is a serious health-related issue that provokes secondary diseases; graft rejection leads to fatalities. Ag nanoparticle coating on the Ti implant and hydroxyapatite facilitates human cells’ biocompatibility with antibacterial efficacy [1].

Previously, Ag nanoparticles were synthesized with hydroxyapatite by the microwave method [47] was very effective for biofilm-forming bacteria and also biocompatible with fibroblast cells, respectively. This indicates that the preparation of nanoparticles and coatings plays a vital role in their applications. A method called FCVA and magnetron sputtering involves the uniform distribution of nanoparticles on the surface and enhances their surface hydrophobicity. Hydrophobicity is a crucial parameter, meaning that the bacterial cells are less likely to attach to the surface. Figure 9 is an illustrative representation of biofilm inhibition on the Ag-taC coatings. The taC coatings on the surface facilitate less adhesion of the cells due to hydrophobicity at a level of 62°, measured by using CA, whereas Ag-doped taC exhibited >75°. It was found that >70° shows good hydrophobicity in nature. Ag in the Ag-taC coatings is in contact with the bacterial cell membranes and releases reactive species to the cells, which target the DNA and proteins necessary for the biosynthesis of molecules, enzymes, and growth substances. These reactive species cause DNA lysis, and eventually cell death occurs. The present study supported the claim that the surface coated with Ag-doped taC did not allow any bacterial cells to adhere to the surface discussed previously (see Figure 7). In contrast, bacterial biofilm was found on the uncoated area of the film. However, additional experiments are required to claim biocompatibility with human cells as the future direction. 

## 4. Conclusions

We summarized the deposition of taC and Ag-taC films on Ti64 alloy, revealed the material’s properties, and optimized the composition, deposition time, and process parameters of FVCA and sputter hybrid PVD systems. The study revealed that Raman spectroscopy, XPS, AFM, and SEM with EDS mapping analysis confirm morphological and structural properties effectively controlled by the doping concentration and conditions. When the plasma discharge was 70 W, the Ar flow rate was 2 cm, the arc current was 60 A, and the substrate bias was −200 V, the fabricated taC film featured a high surface hardness with a uniform and dense texture. In addition, the potential applications of taC and Ag-taC films as coatings on Ti64 alloy were studied with an optimized surface thickness of 200 nm formed at various Ag percentages such as Ag-taC (0.63, 1.34, and 2.01 at. %) for deposition on medical devices and implants. Interestingly, Ag-taC exhibited biofilm formation on the coated Ti64 against *S*. *aureus* and *P. aeruginosa*, which were the primary etiologies for the biofilm-forming bacteria on medical implants. As the concentration of Ag increases in the taC, biofilm formation drastically decreases in the biofilm assay on both pathogens. The bacterial population on the Ag-taC-coated Ti64 was deliberately visualized under SEM studies, which showed that the bacterial biofilm was not found on the coated area, whereas biofilm was observed on the uncoated ones. Hence, based on the presented results, this study confirms that Ag-taC coatings are very effective on devices to overcome biofilm-related medical complications.

## Figures and Tables

**Table 1:** Elemental composition and binding energy of the film estimated through XPS.

Sample	C (at. %)	O (at. %)	Ag (at. %)	Binding Energy (eV)
taC	98.7	1.3	0.0	283.86 (C); 531.38 (O)
Ag-taC (0.63, at %)	95.41	1.47	3.12	284.58 (C); 532.43 (O); 368.26 (Ag)
Ag-taC (1.34, at %)	92.56	1.96	5.48	283.55 (C); 531.45 (O); 363.28 (Ag)
Ag-taC (2.01, at %)	86.66	3.08	10.26	283.57 (C); 531.55 (O); 367.35 (Ag)

## Data Availability

Data will be made available on request.
